# Novel targeted inhibition of the IL-5 axis for drug reaction with eosinophilia and systemic symptoms syndrome

**DOI:** 10.3389/fimmu.2023.1134178

**Published:** 2023-04-28

**Authors:** Limor Rubin, Aviv Talmon, Yaarit Ribak, Asa Kessler, Yossi Martin, Tal Keidar Haran, Oded Shamriz, Irit Adini, Yuval Tal

**Affiliations:** ^1^ Allergy and Clinical Immunology Unit, Department of Medicine, Hadassah Medical Organization, Faculty of Medicine, Hebrew University of Jerusalem, Jerusalem, Israel; ^2^ Department of Medicine, Hadassah Medical Organization, Faculty of Medicine, Hebrew University of Jerusalem, Jerusalem, Israel; ^3^ Psychiatric Department, Hadassah Medical Organization, Faculty of Medicine, Hebrew University of Jerusalem, Jerusalem, Israel; ^4^ Department of Pathology, Hadassah Medical Organization, Faculty of Medicine, Hebrew University of Jerusalem, Jerusalem, Israel; ^5^ The Lautenberg Center for Immunology and Cancer Research, Institute of Medical Research Israel-Canada, Faculty of Medicine, Hebrew University of Jerusalem, Jerusalem, Israel; ^6^ Department of Surgery, Center for Engineering in Medicine and Surgery, Massachusetts General Hospital, Harvard Medical School, Boston, MA, United States

**Keywords:** drug reaction with eosinophilia and systemic symptoms (DRESS), interleukin (IL), monoclonal antibodies (mAbs), IL-5 antibody, biotherapeutic agent

## Abstract

**Background:**

The drug reaction with eosinophilia and systemic symptoms (DRESS) syndrome represents a severe hypersensitivity reaction. Up-to-date treatment is based on withdrawal of medication, supportive care, and immunosuppression using high-dose corticosteroid (CS) therapy. However, evidence-based data are lacking regarding second-line therapy for steroid-resistant or steroid-dependent patients.

**Objectives:**

We hypothesize that the interleukin (IL)-5 axis plays a critical role in the pathophysiology of DRESS; hence, inhibition of this signaling pathway could offer a potential therapy for steroid-dependent and/or steroid-resistant cases, and it may offer an alternative to CS therapy in certain patients more prone to CS toxicity.

**Methods:**

Herein, we collected worldwide data on DRESS cases treated with biological agents targeting the IL-5 axis. We reviewed all cases indexed in PubMed up to October 2022 and performed a total analysis including our center experience with two additional novel cases.

**Results:**

A review of the literature yielded 14 patients with DRESS who were treated with biological agents targeting the IL-5 axis as well as our two new cases. Reported patients are characterized by a female-to-male ratio of 1:1 and a mean age of 51.8 (17–87) years. The DRESS-inducing drugs, as expected from the prospective RegiSCAR study, were mostly antibiotics (7/16), as follows: vancomycin, trimethoprim-sulfamethoxazole, ciprofloxacin, piperacillin-tazobactam, and cefepime. DRESS patients were treated with anti-IL-5 agents (mepolizumab and reslizumab) or anti-IL-5 receptor (IL-5R) biologics (benralizumab). All patients have clinically improved under anti-IL-5/IL-5R biologics. Multiple doses of mepolizumab were needed to achieve clinical resolution, whereas a single dose of benralizumab was often sufficient. Relapse was noted in one patient receiving benralizumab treatment. One patient receiving benralizumab had a fatal outcome, although mortality was probably related to massive bleeding and cardiac arrest due to coronavirus disease 2019 (COVID-19) infection.

**Conclusion:**

Current treatment guidelines for DRESS are based on case reports and expert opinion. Understanding the central role of eosinophils in DRESS pathogenicity emphasizes the need for future implementation of IL-5 axis blockade as steroid-sparing agents, potential therapy to steroid-resistant cases, and perhaps an alternative to CS treatment in certain DRESS patients more prone to CS toxicity.

## 1 Introduction

Drug reaction with eosinophilia and systemic symptoms (DRESS), also known as drug-induced hypersensitivity syndrome, is a severe type of intravenous cutaneous drug-induced eruption accompanied by visceral organ involvement, most commonly the liver. The prognosis of patients with DRESS is linked to the severity of visceral involvement, with an approximate mortality rate of 2%–10% mainly due to liver failure (1).

**Figure 1:**
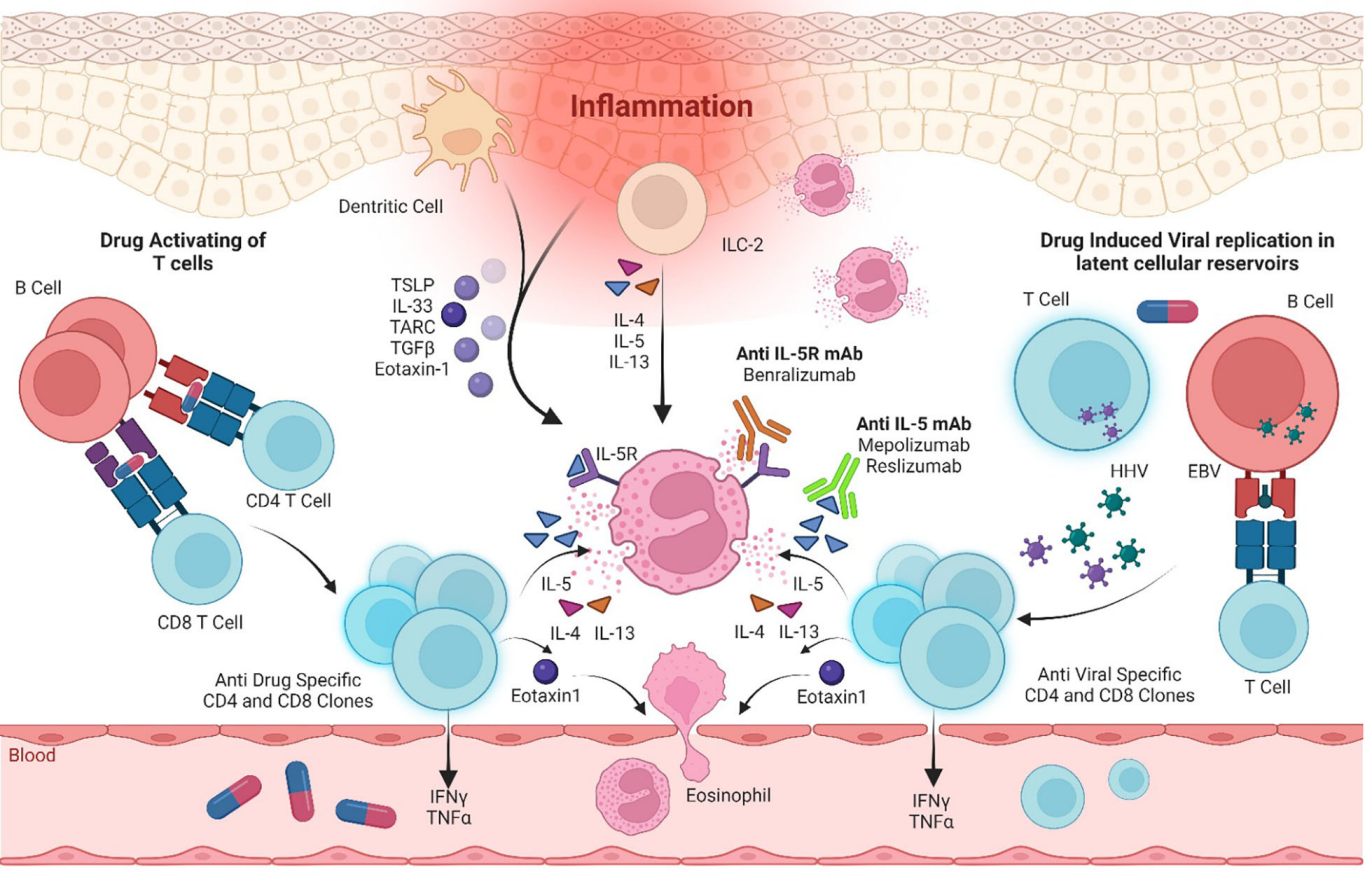
Immunological pathways in DRESS pathogenesis and immunotherapy targeted inhibition of the IL-5 axis. IL, interleukin; IL-5R, interleukin-5 receptor; mAb, monoclonal antibody; EBV, Epstein–Barr virus; HHV, Herpesviridae virus; ILC-2, Type 2 innate lymphoid cells; TSLP, thymic stromal lymphopoietin; TARC, thymus- and activation-regulated chemokine; TGFβ, transforming growth factor β; IFN-γ, interferon γ; TNFα, tumor necrosis factor α.

The pathophysiology is multifactorial and associated with drug metabolism, specific human leukocyte antigen (HLA), and viral reactivation especially of the human Herpesviridae (HHV) family. The immunological response directed against viral reactivation and/or culprit drug precipitating the disease includes CD4^+^ and CD8^+^ T-cell activation and hence the development of a cytokine cascade with the production of interleukin (IL)-5, IL-4, IL-13, IL-17, IL-25, and eotaxin-1 (2). In addition, dermal dendritic cells, endothelial cells, and monomyelocytes secrete thymus activation-regulated chemokine (TARC/CCL17), IL-33, transforming growth factor β, and thymic stromal lymphopoietin. These chemokines, in synergy with IL-5, promote eosinophil chemoattraction, activation, proliferation, and infiltration and hence result in eosinophilic inflammation and tissue damage (3) (**Figure 1**).

Consequently, patients with DRESS present with increased eosinophil levels in the blood, skin, and involved organs. The diagnosis of DRESS is based on clinical and biological criteria as calculated by the RegiSCAR score, such as fever >38°C, acute rash, lymphadenopathy, internal organ involvement, and blood count abnormalities including atypical lymphocytes and eosinophilia, found in 80% of patients (4). There are no randomized trials evaluating treatments for DRESS following the withdrawal of the culprit drug. The current mainstay of treatment is topical and systemic corticosteroids (CSs) (5).

During the last 5 years, limited reports of DRESS patients who were treated with immunotherapy inhibiting the IL-5 axis were presented. Maverakis et al. (6) were the first to describe the potential advantages of anti-IL-5/anti-IL-5 receptor (IL-5R) monoclonal antibodies (mAbs) over current therapies to DRESS patients, emphasizing the rapid onset, once-monthly dosing, and safety, with avoidance of the immunosuppressive and metabolic adverse events of prolonged high-dose systemic CSs. Recently, Gschwend et al. (7) reviewed 14 patients with DRESS who were treated with mAbs inhibiting the IL-5 axis. Patients were reported to have been successfully treated with the anti-IL-5 agents mepolizumab (7–11) and reslizumab (12), as well as the anti-IL-5R agent benralizumab (7, 13–15).

Herein, we aimed to present our experience with two new cases and conduct a literature review as a proof of concept to the hypothesis that IL-5 inhibition offers an efficient and safe therapeutic strategy for specific cases of DRESS.

## 2 Methods

### 2.1 Study design and patients

This is a retrospective analysis of computerized medical records of patients who were diagnosed with DRESS and treated with IL-5 modulation in the period of February to September 2022. Patients were diagnosed and treated at the Division of Medicine, Hadassah Medical Center, Jerusalem, Israel. Data regarding clinical manifestations, treatments, and outcomes were retrieved from the files and analyzed.

### 2.2 Systematic review of the literature

In addition, we conducted a systematic review of the literature concerning patients with DRESS who were treated with IL-5-inhibiting mAbs. We used PubMed for data search. Keywords included “DRESS” and (“IL-5” or “benralizumab” or “mepolizumab” or “reslizumab”). Inclusion criteria consisted of English-language reports of DRESS patients who were treated with mAbs targeting the IL-5 axis and published in the period of May 2018 to October 2022. Only studies in which the DRESS diagnosis was based on the RegiSCAR score were included (**Supplementary Table S1**) (4). This score is available in **Supplementary Table S1**. Studies with a misdiagnosis of DRESS or a limited availability of full text were excluded. Data were analyzed for the clinical presentation, course, and outcome.

### 2.3 Ethical considerations

The reported patients from our medical center have signed a written informed consent for the publication of their clinical data.

## 3 Results

### 3.1 Patient description

**Figure 2:**
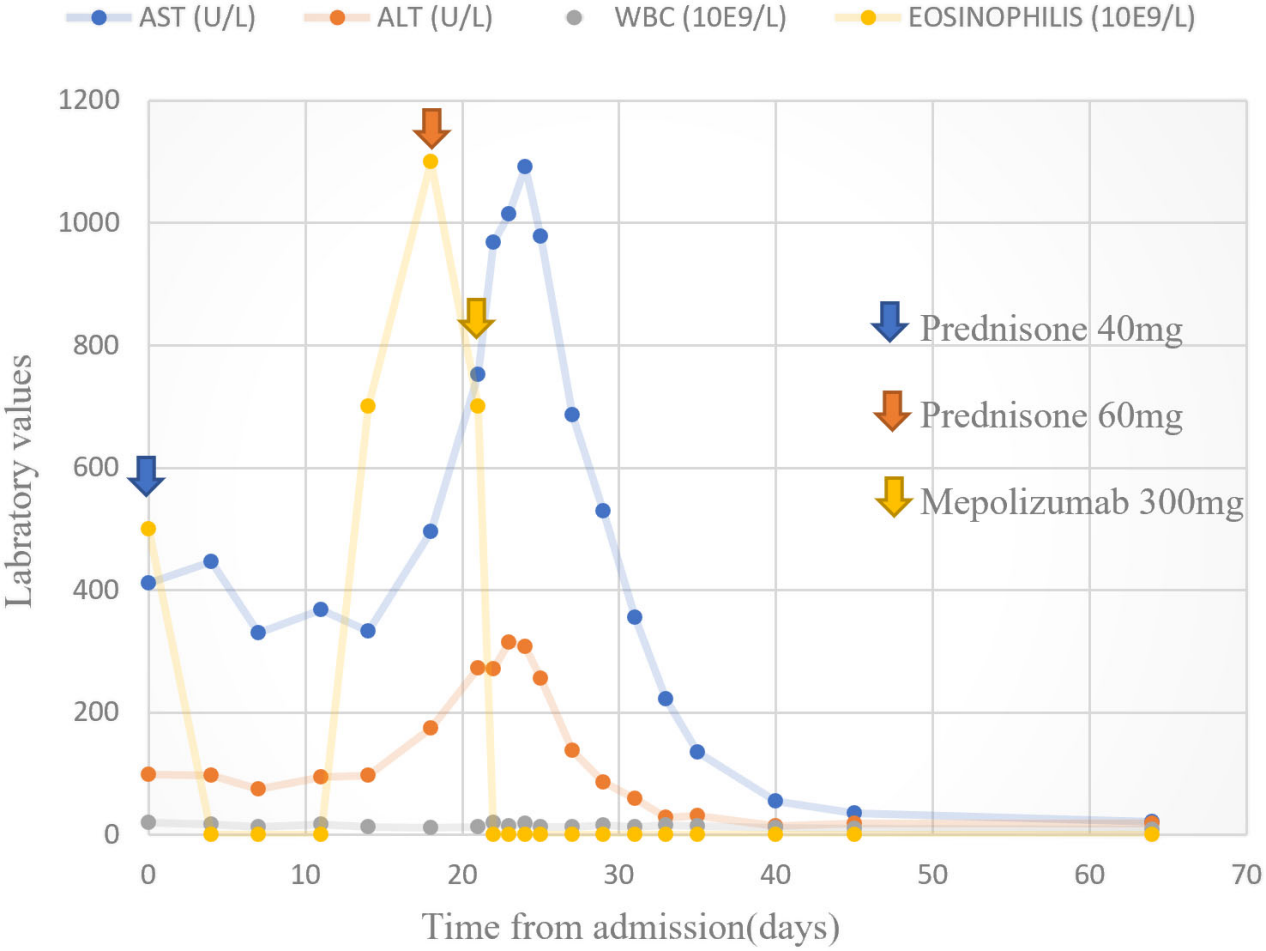
Hepatocellular liver enzymes and absolute eosinophil count. Trend of hepatocellular liver enzymes and absolute eosinophil counts following treatments with prednisone and mepolizumab in patient 1.

**Figure 3:**
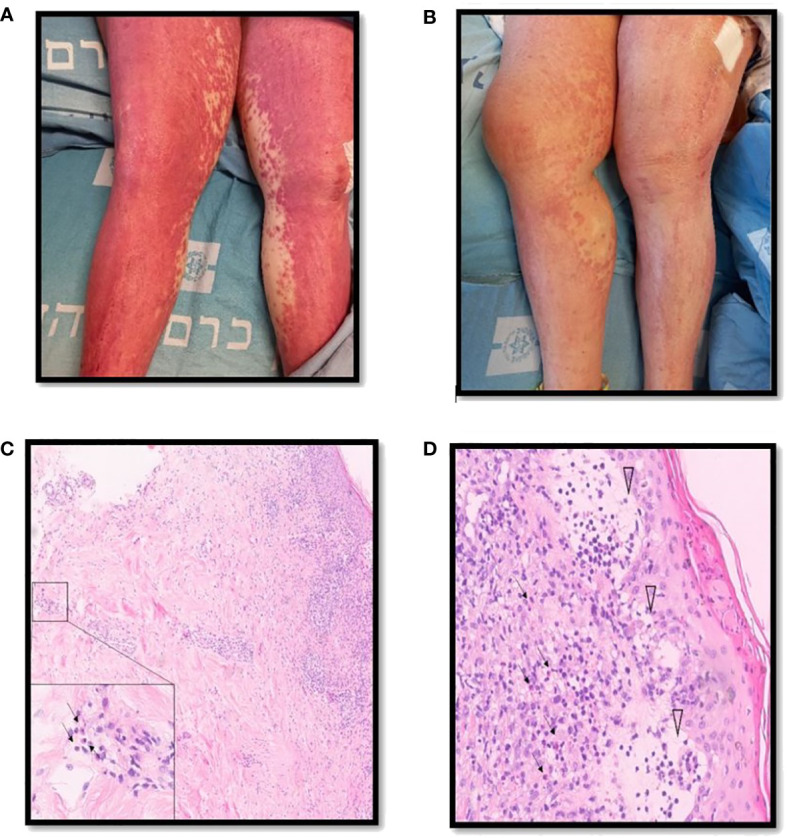
Clinical description and skin histopathology of the patients. **(A)** Clinical presentation of DRESS in patient 2 consisting of diffuse erythematous rash involving the lower limbs. **(B)** One week following benralizumab therapy, partial resolution of the skin rash. **(C)** Punch biopsy of skin consisting of hematoxylin and eosin stain demonstrating changes compatible with the clinical diagnosis of DRESS, most notably an inflammatory infiltrate composed of lymphocytes and numerous eosinophils stretching from the epidermis to the deep dermis in ×10 magnification. The inset demonstrates eosinophils present in the deep dermis in ×100 magnification (arrows). **(D)** Secondary changes including parakeratosis and interstitial edema with blister formation in the papillary dermis (arrowheads) in ×40 magnification.

Our search yielded two female patients (17 and 59 years old) who were admitted to our medical center within the study period. A summary of their clinical characteristics is presented in **Table 1**. Patient (P)1 presented with rash, hepatitis, fever, eosinophilia, and lymphadenopathy induced by olanzapine. She was CS-resistant, and subcutaneous (s.c.) mepolizumab was initiated as a single dose of 300 mg. P2 developed rash, eosinophilia, fever, hepatitis, and acute kidney injury following treatment with vancomycin. She was treated with s.c. benralizumab 30 mg. Both patients have demonstrated clinical resolution of their symptoms following IL-5 inhibitory treatment without recurrence in a follow-up of 10–12 months. The trend of hepatocellular liver enzyme levels and absolute eosinophil counts of P1 following treatments with CSs and mepolizumab is presented in **Figure 2**. The clinical presentation and skin histopathology of P1 and P2 are presented in **Figure 3**. The full clinical and laboratory description of the two patients is presented in the **Supplementary Material**.

**Table 1:** Clinical characteristics of DRESS patients treated with IL-5-inhibiting biologics.

Patient	Age (years)	Sex	Ethnicity	Medical history	Offending drug	Clinical manifestations of DRESS	RegiSCAR DRESS score	Maximal eosinophil count (cells/ml)	Histopathology	Treatment	IL-5-modulating treatment	Indications for anti-IL-5/IL-5R biologics	Outcome	Follow-up (months)
P1	17	F	J	Psychosis	Olanzapine	Rash, hepatitis, fever, eosinophilia, lymphadenopathy	6	1,620	Skin biopsy suggestive of DRESS	CS	Mepolizumab 300 mg, s.c. single dose	CS-resistant, avoid steroid toxicity	Complete resolution of relapsing DRESS syndrome and complete weaning from CSs	12
P2	59	F	J	HTN	Vancomycin	Rash, eosinophilia, fever, hepatitis, acute kidney injury	7	1,600	Skin biopsy suggestive of DRESS	CS	Benralizumab 30 mg s.c. single dose	First-line treatment to avoid steroid toxicity	Complete resolution of relapsing DRESS syndrome and complete weaning from CSs	10

F, female; J, Jew; HTN, hypertension; anti-IL-5R, anti-interleukin-5 receptor; CSs, corticosteroids; s.c., subcutaneous.

### 3.2 Literature review

A review of the literature yielded 14 patients with DRESS who were treated with biological agents targeting the IL-5 axis (**Table 2**). The first case was reported on May 2018. Nine studies that met the inclusion criteria (7–15) and our two clinical cases were included in the analysis. The reported patients are characterized by a female-to-male ratio of 1:1 and a mean age of 51.8 (17–87) years. The DRESS-inducing drugs, as expected from the prospective RegiSCAR study, were mostly antibiotics (7/16), as follows: vancomycin, trimethoprim-sulfamethoxazole, ciprofloxacin, piperacillin-tazobactam, and cefepime. The DRESS patients were treated with anti-IL-5 agents (mepolizumab and reslizumab) or anti-IL-5R biologics (benralizumab).

**Table 2:** Clinical characterizations of the DRESS patients treated off-label with monoclonal antibodies of the IL-5 axis.

Biological therapy/mechanism	No. of patients	Gender	Age (years)	RegiSCAR DRESS score ^a^	Histopathology	Drug inducing DRESS	IL-5 axis treatment regimen (Indication)	Additional combined, adjunct treatment	Outcome	Ref.
**Reslizumab/** **/anti-IL-5 IgG4κ monoclonal antibody**	1	F	62	3	NA	Imatinib	Initial dose 100 mg i.v. 2 weeks later, a second dose of 200 mg i.v. (CS-dependent, desensitization)	Prednisolone, antihistamine Dexamethasone **^g^**	Complete resolution of symptoms. Reslizumab enabled desensitization to safely maintain imatinib treatment for 2 years.	(12)
**Mepolizumab** **/anti- IL-5 IgG1κ monoclonal antibody**	1	F	17	6	**Skin Biopsy**- Suggestive of DRESS	Olanzapine	Single loading dose 300 mg s.c. (CS-dependent)	Hydrocortisone followed by prednisone	Complete resolution of relapsing DRESS syndrome and complete weaning from CSs; **f/o-** 6 months symptom-free	Current report
	1	F	56	6	**Skin biopsy-** a spongiotic reaction pattern, spongiotic vesicles, small pustules with lymphocyte, and eosinophil predominance; **Bone marrow biopsy-** reactive, hypercellular marrow with marked eosinophilia	Trimethoprim-sulfamethoxazole	100 mg s.c., monthly, for 3 months (CS-dependent)	Dexamethasone and IVIG, followed by a reduced dose of prednisone **^c^**	Complete resolution of relapsing DRESS syndrome and complete weaning from CSs; 6-month follow-up – no recurrence	(8)
	1	M	56	6	NA	Pregabalin	**Initial dose-** 600 mg, s.c. split to 300 mg on consecutive days; **Maintenance dose**-300 mg, s.c. every 4 weeks (CS-resistant)	Methylprednisolone followed by prednisolone **^d^**	Rapid effect on eosinophil count and resolution of pulmonary symptoms in a patient with acute pneumonitis secondary to DRESS **f/o**- readmission due to fibrotic sequelae.	(9)
		M	50s	5	**Skin biopsy**- urticarial pustular reaction pattern, minor interface change, the presence of eosinophils **Myocardium biopsy-** lymphohistiocytic and eosinophilic infiltrate with no necrosis	Ciprofloxacin	2 doses of 300 mg s.c. with a 3-week interval (CS-resistant myocarditis)	Oral prednisolone, methylprednisolone 3 pulses, followed by cyclophosphamide and cyclosporine **^e^**	The patient completed a 6-month course of cyclophosphamide without complications and his prednisolone and cyclosporine have both been entirely weaned with complete clinical and laboratory recovery and no features of cardiac dysfunction	(10)
		F	45	NA	**Myocardium biopsy-** dense eosinophilic infiltrates and myocardial necrosis with few giant cell	Lamotrigine	300 mg i.v. monthly for 3 months followed by 500 mg monthly up to 1 year (CS-dependent myocarditis)	Methylprednisolone, mycophenolate mofetil, colchicine, cyclosporine, followed by a reduced dose of prednisone **^f^**	Patient has remained asymptomatic and discontinued her mycophenolate, taper slowly prednisone. **f/o** - 1 year symptom-free	(11)
		M	62	7	NA	Amoxicillin	Three doses of 100 mg s.c. (CS-resistant)	Systemic high-dose CSs*	Rapid improvement, complete remission of DRESS and weaning from CSs	(7)
		M	70	6	NA	Piperacillin/vancomycin/meropenem	Single dose of 100 mg s.c. (To avoid CS toxicity- during sepsis)	Systemic CSs*	Minor improvement, possibly because TEN/DRESS overlap	(7)
**Benralizumab** **/anti-IL-5 receptor IgG1κ monoclonal antibody**		F	59	7	**Skin Biopsy-** Suggestive of DRESS	Vancomycin	Single loading dose 30 mg s.c. (First-line treatment to avoid CS toxicity)	Hydrocortisone 100 mg followed with prednisone 40 mg	Complete resolution of relapsing DRESS syndrome and complete weaning from CSs; **f/o –** 4 m symptom-free	Current report
		F	54	7	**Skin Biopsy**- Perivascular, lymphohistiocytic infiltrate, and eosinophils.	Esomeprazole, piperacillin-tazobactam	Single dose 30 mg s.c. (First-line treatment, CS-resistant)	Methylprednisolone **^h^**	Clinical and laboratory improvement over the following 18 days	(13)
		M	58	8	**Skin Biopsy**- Perivascular, lymphohistiocytic infiltrate, and eosinophils.	Midazolam	Single dose 30 mg s.c. (First-line treatment, CS-resistant)	Methylprednisolone **^i^**	Cutaneous improvement, decline in liver function tests and eosinophil levels; developed disseminated intravascular coagulation secondary to COVID-19 and died from cardiac arrest after massive bleeding 17 days later.	
		F	87	5	**Skin Biopsy**-Suggestive of DRESS	Allopurinol- pregabalin	Single dose 30 mg s.c. (CS-resistant)	Methylprednisolone followed by prednisone **^j^**	Rapid and complete clinical recovery (defined as regression of cutaneous-systemic symptoms and eosinophilia) with concurrent tapering of low-dose GC	(15)
		M	74	6	**Skin Biopsy**-Suggestive of DRESS	Allopurinol	Single dose 30 mg s.c. (CS-resistant)	Methylprednisolone followed by prednisone **^j^**		
		F	67	7	**Skin Biopsy**-Suggestive of DRESS	Ibuprofen-paracetamol	Single dose 30 mg s.c. followed by two doses of mepolizumab 100 mg s.c. (4-week interval) (CS-resistant)	Methylprednisolone followed by prednisone **^j^**	Long relapsing course of DRESS before starting benralizumab, clinical relapse and eosinophilia 4 months after the injection and mepolizumab was initiated. **f/o**- symptom-free under treatment	
		M	43	8	**Skin biopsy**- eczematiform toxidermia; **Blood smear-** severe hemophagocytic lymphohistiocytosis with profound thrombocytopenia	Cefepime	2 doses of 30 mg s.c. with a 4-week interval (CS-resistant)	Topical CSs, methylprednisolone and IVIG followed by prednisone **^k^**	Decreased eosinophilia within 2 days, resolution of hemophagocytic lymphohistiocytosis, and improvement in both organ dysfunction and skin lesions	(14)
		M	39	6	**Liver biopsy** - eosinophilic hepatitis	Metamizole	3 doses of 30 mg s.c. at 4-week intervals (CS-resistant)	Prednisone*	Rapid suppression of eosinophils and hepatitis improved. CS could be reduced to under 10 mg prednisolone after 7 months.	(7)

M, male; F, female; CSs, corticosteroids; ANEM, acute necrotizing eosinophilic myocarditis; IVIG, intravenous immunoglobulin; s.c., subcutaneous injection; i.v., intravenous injection; NA, data are not available.

**^a^** According to RegiSCAR DRESS validation scoring system criteria; cutoff points include the following: suspected cases as definite (scores 6–8), probable (scores 4–5), possible (scores 2–3), and no DRESS (score <2).

**^b^** One patient in addition to benralizumab received two doses of mepolizumab 100 mg (4-week interval).

**^c^** Dexamethasone 10 mg four times a day and i.v. IgG 70 g (1 g/kg) for 2 days; after clinical improvement, she was started on prednisone 70 mg daily and then was weaned by 10 mg fortnightly; two and a half weeks later, prednisone was reduced to 60 mg daily; after DRESS syndrome diagnosis, prednisone dose was increased to 110 mg per day for 2 days with good response; within 3 days of the first dose given together with mepolizumab, prednisone dose was reduced to 10 mg weekly, until 20 mg over the next 6 weeks, and thereafter it was tapered at a slower rate until it was ceased completely in 3 months.

**^d^** Upon DRESS diagnosis i.v. methylprednisolone (1 g daily for 6 days), followed by prednisolone 60 mg daily due to progression of DRESS symptoms Mepolizumab was administered.

**^e^** Initially, 75 mg oral prednisolone 5 days and tapering down, following diagnosis of myocarditis a 3-day course of i.v. 250 mg methylprednisolone was administered, with transition to oral prednisolone 1 mg/kg. However, due to troponin elevation, another 3-day course of 500 mg methylprednisolone followed by a 3-day course of methylprednisolone 1 g combined treatment of mepolizumab with 6-month course of monthly intravenous 1,500 mg cyclophosphamide; 3 weeks following mepolizumab injection troponinemia–another dose of mepolizumab and CS pulse.

**^f^** Methylprednisolone 500 mg two times per day and mycophenolate mofetil 1,000 mg two times per day in addition to colchicine 0.6 mg two times per day, followed by prednisone 60 mg daily, and cyclosporine 100 mg two times per day, upon tapering to 30 mg prednisone relapse-with mepolizumab and slow taper of prednisone at 2.5 mg.

**^g^** Oral prednisolone 15 mg was imitated prior to DRESS diagnosis, antihistamines were administered during desensitization and intravenous dexamethasone was given before, but not after reslizumab.

**^h^** Methylprednisolone 125 mg for 3 days, 70 mg for 4 days followed by benralizumab due to clinical deterioration.

*CS dose or type of agent not reported.

**^i^** Methylprednisolone 125 mg for 3 days followed by benralizumab due to clinical deterioration.

**^j^** Methylprednisolone 125 mg for 3–4 days followed by 40–60 mg prednisolone, a single administration of benralizumab and concurrent tapering of CS.

**^k^** Methylprednisolone i.v. 2 mg/kg/day, then 1 g/day for 3 days and IVIG 1 g/day for 2 days.

#### 3.2.1 Reslizumab

Reslizumab was administered in one 62-year-old woman with imatinib-induced DRESS (12). Her RegiSCAR DRESS score was 3, and histopathology was not available when confirming the DRESS diagnosis. Reslizumab treatment has enabled complete resolution of the patient’s symptoms, facilitated desensitization, and succeeded in safely maintaining the drug imatinib treatment for 2 years.

#### 3.2.2 Mepolizumab

Six DRESS patients were treated with mepolizumab (7–11). The mean (range) age of the patients was 50.8 (17–70) years. The RegiSCAR score calculated ranged between 5 and 7. Four out of seven patients had histopathologic evidence supporting eosinophilic inflammation. Five out of seven DRESS patients treated with mepolizumab need two or more doses, and only two have clinically improved following a single dose. All patients received concurrent CS therapy, one received additional therapy with intravenous immunoglobulin (IVIG), and two received concurrent immunosuppressant therapy with cyclosporine and one with cyclophosphamide. The clinical indication for the initiation of anti-IL-5-targeted therapy was due to steroid resistance and dependence in three and two patients, respectively. In one patient, IL-5-blocking agents were initiated to avoid CS toxicity.

All patients responded with complete resolution of DRESS symptoms, laboratory recovery, and complete weaning off from steroid therapy. Relapse was noted in one patient.

#### 3.2.3 Benralizumab

Seven DRESS patients were treated with benralizumab (7,13–15). The mean patient age was 68.7 (34–87) years. The RegiSCAR score ranged from 5 to 8. All patients receiving benralizumab had histopathology supporting tissue eosinophilic infiltration. The indication for benralizumab initiation was CS resistance in all patients. Two patients required two or more doses. Relapse was noted in one patient receiving benralizumab treatment (15). Moreover, one patient had a fatal outcome, although mortality was probably related to massive bleeding and cardiac arrest due to coronavirus disease 2019 (COVID-19) infection (13).

## 4 Discussion

DRESS syndrome is a rare life-threatening hypersensitivity reaction; hence, scarce data exist regarding treatment protocols. Data presented in our study, including two unreported patients from our medical center, support treatment of DRESS with mAbs directed toward IL-5 or IL-5R, which are already Food and Drug Administration (FDA)-approved for other eosinophilic disorders.

IL-5 plays a crucial role in eosinophilic pathophysiology and is proposed as a novel therapeutic target for hypereosinophilic syndrome and rare eosinophilic conditions (16, 17). This cytokine is the major differentiation factor for eosinophils, playing a pivotal role in innate and acquired immune responses and eosinophilia (18). The IL-5R is a heterodimer comprising one alpha subunit (IL-5Rα) and one beta subunit (IL-5Rβ) that, upon activation by IL-5 signals, stimulate the Janus kinase (JAK)–signal transducer and activator of transcription proteins (STATs) pathway (19). Therefore, the reduction of blood eosinophil levels by antagonizing IL-5 and its receptor using mAbs recently becomes an important immunotherapeutic strategy (**Figure 1**).

Second-line therapy for patients with DRESS and severe organ involvement who do not respond to systemic CSs or for patients in whom CSs are contraindicated includes cyclosporine, IVIG, cyclophosphamide, and the JAK inhibitor tofacitinib, despite evidence of high failure rates, relapse, and excess of adverse events including serious infections (20–23).

With novel targeted biological agents and a better understanding of the key role of the IL-5 axis in DRESS, there are case reports of treatment with anti-IL-5 or anti-IL-5R mAbs, such as mepolizumab, reslizumab, or benralizumab. Mepolizumab is an anti-IL-5 humanized IgG1κ antibody that is FDA-approved for the treatment of severe eosinophilic asthma, hypereosinophilic syndrome, and eosinophilic granulomatosis with polyangiitis (EGPA) (24). Reslizumab is an anti-IL-5 humanized IgG4κ antibody with FDA approval for severe eosinophilic asthma (25), and benralizumab is a humanized fucosylated IgG1κ anti-IL-5Rα antibody approved by the FDA for the treatment of severe eosinophilic asthma (26).

Two previously published studies involving large cohorts reported treatment with anti-IL-5 agents in DRESS patients. In a European international multicenter cohort, Kridin et al. (27) identified four DRESS patients who were treated with anti-IL-5 biologics. DRESS patients treated with anti-IL-5/IL-5R agents were mostly CS-refractory cases who had longer hospitalizations, increased rates of intensive care unit admissions, and a higher risk of relapses (27). Gschwend et al. (7) summarized 14 DRESS patients treated with anti-IL-5 agents. While treatment with reslizumab or mepolizumab appeared to require repeated doses to achieve clinical resolution in most patients, a single dose of benralizumab was shown to be sufficient, thus indicating that treatment with benralizumab is more efficient than that with mepolizumab or reslizumab (7).

P1 in our report emphasizes the complexity in the initiation and prolonged administration of CS therapy in the psychiatric patient due to its adverse effects (28). This also stresses the role of therapy with an IL-5-/IL-5R-targeting mAbs as a CS-sparing agent. Gschwend et al. (7) recommended using anti-IL-5/IL-5R biologics in specific DRESS patients including those with a severe course, CS-resistant disease, severe disease with concomitant infection, or severe end-organ damage at presentation. P1 further expands these criteria, as her psychiatric disorder indicated the need for a CS-sparing treatment. This can be further implemented on DRESS patients with underlying disorders that constitute relative or absolute contraindications for prolonged CS treatment, such as uncontrolled diabetes. On the other hand, P2 emphasizes the role of early initiation of therapy with an IL-5-/IL-5R-targeting mAb to avoid CS toxicity, as the CS effect on bone reabsorption is a secondary insult to a patient presenting with a pathologic bone fracture from osteomyelitis (29).

Systemic CSs are still considered to be the mainstay of treatment for DRESS. Early initiation of a steroid-sparing agent is vital to reduce CS side effects. Cumulatively, as evident from our patients and all others reviewed (**Table 2**) (7, 30), it is evident that therapy with mAbs directed toward the IL-5/IL-5R axis combined with adjunct treatments could offer an alternative to CS therapy in some patients. DRESS patients eligible to receive anti-IL-5/IL-5R mAbs include those recommended by Gschwend et al. (7) and other patients with a high risk for CS toxicity including patients with psychiatric disorders or comorbidities such as metabolic disorders, immunocompromised patients, and the elderly.

This study presents several limitations, mainly its retrospective design and small number of patients. Furthermore, the characteristics of patients such as age and sex and drug treatments differed among the different studies. A multicenter study with a larger sample size is required for prolonged follow-up and investigation of the proper dose regimen of anti-IL-5/IL-5R treatment for DRESS. DRESS often shows severe symptoms in the acute phase with serious disease sequelae in the chronic phase. Therefore, careful follow-up is required. Since there is no definite view on how to treat and which regimen to follow, the use of mAbs directed toward the IL-5/IL-5R axis is an important emerging issue in DRESS therapy deserving further clinical investigations.

In conclusion, future implementation of mAbs directed toward the IL-5/IL-5R axis in DRESS cases presents a promising therapeutic strategy in DRESS patients. Selected DRESS patients eligible to receive anti-IL-5-/IL-R-blocking agents as first-line treatment consist of patients with contraindications to CS therapy, while the risk of relapse may still exist. IL-5-modulating agents can also be used in DRESS patients with a CS-dependent or -resistant clinical course. Thus, anti-IL-5/IL-5R biologics may offer a novel therapeutic modality in these patients.

## Data availability statement

The raw data supporting the conclusions of this article will be made available by the authors, without undue reservation.

## Ethics statement

Written informed consent was obtained from the individual(s) for the publication of any potentially identifiable images or data included in this article.

## Author contributions

LR: treatment of patients and writing of the manuscript; AT: mansucruipt revisions and figure design, YR, AK, and YM: manuscript revisions and treatment of patients; TH: pathology workup; IA: manuscript revisions; OS and YT: correspocnding authors and manuscript design and revisions. All authors contributed to the article and approved the submitted version.

## References

[1] Ichai P Laurent-Bellue A Saliba F Moreau D Besch C Francoz C. Acute liver Failure/Injury related to drug reaction with eosinophilia and systemic symptoms: outcomes and prognostic factors. Transplantation (2017) 101(8):1830–7. doi: 10.1097/TP.0000000000001655 28207633

[2] Musette P Janela B. New insights into drug reaction with eosinophilia and systemic symptoms pathophysiology. Front Med (Lausanne) (2017) 4:179. doi: 10.3389/fmed.2017.00179 29255708 PMC5722807

[3] Ganeshanandan L Lucas M. Drug reaction with eosinophilia and systemic symptoms: a complex interplay between drug, T cells, and herpesviridae. Int J Mol Sci (2021) 22(3):1127. doi: 10.3390/ijms220311271127 33498771 PMC7865935

[4] Kardaun SH Sekula P Valeyrie-Allanore L Liss Y Chu CY Creamer D. Drug reaction with eosinophilia and systemic symptoms (DRESS): an original multisystem adverse drug reaction. results from the prospective RegiSCAR study. Br J Dermatol (2013) 169(5):1071–80. doi: 10.1111/bjd.12501 23855313

[5] Martinez-Cabriales SA Rodriguez-Bolanos F Shear NH. Drug reaction with eosinophilia and systemic symptoms (DReSS): how far have we come? Am J Clin Dermatol (2019) 20(2):217–36. doi: 10.1007/s40257-018-00416-4 30652265

[6] Maverakis E Ji-Xu A Bruggen MC. Targeting interleukin-5 with benralizumab: a novel treatment for drug rash with eosinophilia and systemic symptoms. Allergy (2022) 77(8):2287–9. doi: 10.1111/all.15283 35285038

[7] Gschwend A Helbling A Feldmeyer L Mani-Weber U Meincke C Heidemeyer K. Treatment with IL5-/IL-5 receptor antagonists in drug reaction with eosinophilia and systemic symptoms (DRESS). Allergo J Int (2022), 1–8. doi: 10.1007/s40629-022-00224-7 PMC9396594 36035809

[8] Ange N Alley S Fernando SL Coyle L Yun J. Drug reaction with eosinophilia and systemic symptoms (DRESS) syndrome successfully treated with mepolizumab. J Allergy Clin Immunol Pract (2018) 6(3):1059–60. doi: 10.1016/j.jaip.2017.10.020 29133221

[9] Thein OS Sutton B Thickett DR Parekh D. Mepolizumab rescue therapy for acute pneumonitis secondary to DRESS. BMJ Case Rep (2019) 12(10). doi: 10.1136/bcr-2019-231355 PMC6803131 31604720

[10] Truong K Kelly S Bayly A Smith A. Successful mepolizumab treatment for DRESS-induced refractory eosinophilic myocarditis and concurrent thyroiditis. BMJ Case Rep (2021) 14(7). doi: 10.1136/bcr-2021-242240e24224014/7/e242240 PMC8286746 34266818

[11] Kowtoniuk R Pinninti M Tyler W Doddamani S. DRESS syndrome-associated acute necrotizing eosinophilic myocarditis with giant cells. BMJ Case Rep (2018), 2018. doi: 10.1136/bcr-2018-22646bcr2018226461 PMC6194447 30301732

[12] Park H Choi GS Lee EM. Successful treatment of imatinib-induced DRESS syndrome using reslizumab without cessation of imatinib: a case report. Case Rep Oncol (2021) 14(3):1548–54. doi: 10.1159/000519471cro-0014-1548 PMC8613629 34899250

[13] Schmid-Grendelmeier P Steiger P Naegeli MC Kolm I Claudia Cecile Valerie L Maverakis E. Benralizumab for severe DRESS in two COVID-19 patients. J Allergy Clin Immunol Pract (2021) 9(1):481–483.e2. doi: 10.1016/j.jaip.2020.09.039 33039646 PMC7543785

[14] Mesli F Dumont M Soria A Groh M Turpin M Voiriot G. Benralizumab: a potential tailored treatment for life-threatening DRESS in the COVID-19 era. J Allergy Clin Immunol Pract (2021) 9(9):3529–3531.e1. doi: 10.1016/j.jaip.2021.06.047 PMC8279918 34273579

[15] Lang CCV Schmid-Grendelmeier P Maverakis E Bruggen MC. Reply to "Benralizumab: a potential tailored treatment for life-threatening DRESS in the COVID-19 era". J Allergy Clin Immunol Pract (2021) 9(9):3531–2. doi: 10.1016/j.jaip.2021.06.048 PMC8299284 34273580

[16] Harish A Schwartz SA. Targeted anti-IL-5 therapies and future therapeutics for hypereosinophilic syndrome and rare eosinophilic conditions. Clin Rev Allergy Immunol (2020) 59(2):231–47. doi: 10.1007/s12016-019-08775-4 31919743

[17] Shamriz O Hershko AY Talmon A Ribak Y Elazary AS Horev L. The efficacy of off-label IL-5-modulating treatment in rare eosinophil-mediated diseases. Allergol Int (2021) 70(2):266–8. doi: 10.1016/j.alit.2020.10.001 33779559

[18] Kouro T Takatsu K. IL-5- and eosinophil-mediated inflammation: from discovery to therapy. Int Immunol (2009) 21(12):1303–9. doi: 10.1093/intimm/dxp102dxp102 19819937

[19] Broughton SE Nero TL Dhagat U Kan WL Hercus TR Tvorogov D. The betac receptor family - structural insights and their functional implications. Cytokine (2015) 74(2):247–58. doi: 10.1016/j.cyto.2015.02.005 25982846

[20] Joly P Janela B Tetart F Rogez S Picard D D'Incan M. Poor benefit/risk balance of intravenous immunoglobulins in DRESS. Arch Dermatol (2012) 148(4):543–4. doi: 10.1001/archderm.148.4.dlt120002-c 22508885

[21] Kim D Kobayashi T Voisin B Jo JH Sakamoto K Jin SP. Targeted therapy guided by single-cell transcriptomic analysis in drug-induced hypersensitivity syndrome: a case report. Nat Med (2020) 26(2):236–43. doi: 10.1038/s41591-019-0733-7 PMC7105105 31959990

[22] Laban E Hainaut-Wierzbicka E Pourreau F Yacoub M Sztermer E Guillet G. Cyclophosphamide therapy for corticoresistant drug reaction with eosinophilia and systemic symptoms (DRESS) syndrome in a patient with severe kidney and eye involvement and Epstein-Barr virus reactivation. Am J Kidney Dis (2010) 55(3):e11–4. doi: 10.1053/j.ajkd.2009.10.054 20110143

[23] Nguyen E Yanes D Imadojemu S Kroshinsky D. Evaluation of cyclosporine for the treatment of DRESS syndrome. JAMA Dermatol (2020) 156(6):704–6. doi: 10.1001/jamadermatol.2020.0048 PMC7066519 32159726

[24] Pavord ID Bel EH Bourdin A Chan R Han JK Keene ON. From DREAM to REALITI-a and beyond: mepolizumab for the treatment of eosinophil-driven diseases. Allergy (2022) 77(3):778–97. doi: 10.1111/all.15056 PMC9293125 34402066

[25] Hashimoto S Kroes JA Eger KA Mau Asam PF Hofstee HB Bendien SA. Real-world effectiveness of reslizumab in patients with severe eosinophilic asthma - first initiators and switchers. J Allergy Clin Immunol Pract (2022) 10(8):2099–2108.e6. doi: 10.1016/j.jaip.2022.04.014 35487369

[26] Zhu M Yang J Chen Y. Efficacy and safety of treatment with benralizumab for eosinophilic asthma. Int Immunopharmacol (2022) 111:109131. doi: 10.1016/j.intimp.2022.109131 35998507

[27] Kridin K Bruggen MC Walsh S Bensaid B Ranki A Oppel E. Management and treatment outcome of DRESS patients in Europe: an international multicentre retrospective study of 141 cases. J Eur Acad Dermatol Venereol (2023) 37(4):753–62. doi: 10.1111/jdv.18808 36479739

[28] Dubovsky AN Arvikar S Stern TA Axelrod L. The neuropsychiatric complications of glucocorticoid use: steroid psychosis revisited. Psychosomatics (2012) 53(2):103–15. doi: 10.1016/j.psym.2011.12.007 22424158

[29] Lane NE. Glucocorticoid-induced osteoporosis: new insights into the pathophysiology and treatments. Curr Osteoporos Rep (2019) 17(1):1–7. doi: 10.1007/s11914-019-00498-x 30685820 PMC6839409

[30] Pitlick MM Li JT Pongdee T. Current and emerging biologic therapies targeting eosinophilic disorders. World Allergy Organ J (2022) 15(8):100676. doi: 10.1016/j.waojou.2022.100676100676 35983569 PMC9356173

