# Supplementary material for: Novel targeted inhibition of the IL-5 axis for drug reaction with eosinophilia and systemic symptoms syndrome
Source: Front Immunol. 2023 Apr 28;14:1134178. doi: 10.3389/fimmu.2023.1134178 (PMC10175640; doi:10.3389/fimmu.2023.1134178)
Supplement: Supplementary file 1 [file DataSheet_1.docx]

Supplementary Material

# Supplementary Data

A retrospective analysis of patients, diagnosed with DRESS and treated at Hadassah Medical Center, Jerusalem, Israel in the period of February to September 2022 yielded 2 female patients (17 and 59 years old).

# Patient description

**1. 1 – Patient 1**

Patient (P)1 presented to the emergency room (ER) with an acute psychotic attack. The patient was admitted to the psychiatric department, and antipsychotic treatment was initiated with 10 mg olanzapine and 2 mg lorazepam. A month later, the dose of olanzapine was increased to 20 mg, and 10 days following the dose change, the patient developed a diffused maculopapular rash, dryness, and peeling of the skin, accompanied by fever (38.5 ºC). The patient arrived at the ER, laboratory tests revealed elevated liver function test (LFT), aspartate transaminase (AST) 95 U/L and alanine transaminase (ALT) 153/U/L, with no eosinophilia. Infectious workup was negative for mycoplasma, Strep. group A throat culture, and viral serology for Epstein-Barr virus (EBV), Cytomegalovirus (CMV), human immunodeficiency virus (HIV), Hepatitis A virus (HAV), Hepatitis B virus (HBV), and Hepatitis C virus (HCV). The patient was discharged with empirical antibiotic treatment with penicillin G and azithromycin.

One-day following discharge, upon exacerbation, the patient returned to the ER with a diffuse maculopapular rash, facial puffiness, fever, and low blood pressure (70/40 mmHg). The patient was admitted to the intensive care unit (ICU), with a working diagnosis of septic shock. Intravenous antibiotic with ceftriaxone and clindamycin was initiated, in addition to fluid resuscitation and vasopressor support with noradrenaline. During her hospitalization, blood cultures were negative, eosinophilia raised up to 1620 x10^9^/L, and her LFT were increased with AST of 68 U/L and ALT 245 U/L.

The patient underwent a head computerized tomography (CT), lumbar puncture, and an electroencephalogram (EEG), which were all unremarkable. Skin biopsy was undertaken with pathology results consisting of the diagnosis of DRESS. Hence, IV hydrocortisone 100 mg, 3 times a day, and psychiatric treatment with perphenazine 8 mg, twice a day, were initiated with clinical and laboratory improvement. The patient was transferred to the tertiary center psychiatric department a month later, with prednisone 40 mg, twice a day, in addition to topical CS. Upon her admission, due to clinical and laboratory improvement, the patient started tapering down CS dose to 40 mg, once a day. Due to psychiatric instability, electroconvulsive therapy (ECT) was initiated in addition to psychiatric treatment with perphenazine 8 mg, and biperiden 2 mg. Following tapering down of CS, upon reaching prednisone 30 mg, the patient had a relapse with the reappearance of skin rash, elevation of eosinophils levels up to 1100 x 10^9^/L, and elevation of LFT. The patient underwent a negative hepatitis workup- viral and immunological serology, and an abdomen Doppler ultrasound. Due to steroid exacerbation of psychosis, and with the goal to induce a steroid-sparing agent following informed consent and committee approval of off-label drug administration, mepolizumab 300 mg, single dose was administered s.c. Three days following therapy the patient improved, the rash disappeared, and LFT and eosinophil declined significantly (Supplement Fig. 1) and in one month, the patient managed to taper-down steroids completely. The patient’s psychiatric disorder improved upon steroid withdrawal and initiation of ECT. Out-clinic 6 months follow-up following mepolizumab therapy was unremarkable, with no clinical symptoms or laboratory abnormalities.

**1. 2 - Patient 2**

P2 had a past medical history of hypertension, stable on ramipril treatment, was admitted to ER complaining of left thigh pain. CT scan revealed a large collection of fluid surrounding the left femur, measuring 125 x 48 x 91 mm. The patient underwent surgical drainage of bloody pus sent to microbiological cultures positive to *Streptococcus intermedius*, intermediate sensitive to cephalexin. IV cefazolin antibiotic, 6 gram per day, was initiated. Thus, a peripheral central catheter line was inserted to the patient and she was discharged to continue treatment course.

One month later, the patient presented again to the ER with left thigh pain. CT scan revealed a mid-shaft femur fracture and an adjacent large collection. She underwent drainage and surgical fixation of the fracture with a gentamicin-coated nail. Bacterial cultures revealed Methicillin-resistant *Staphylococcus epidermidis* and hence vancomycin was initiated. Three weeks later, she presented to the ER with recurrent left thigh pain and high-grade fever. CT scan demonstrated a large collection; she was admitted to the orthopedic department and underwent surgical drainage, with negative cultures. Treatment with vancomycin was continued with a dose increase due to low trough drug level. One week following dose escalation, she was febrile (38.5 ºC) and developed a diffuse erythematous rash involving the chest, back, abdomen, limbs and palms with no mucosal involvement, and facial puffiness with periorbital angioedema (Supplement Fig.2 A,B). Blood tests revealed acute kidney injury, eosinophilia up to 1600 x 10^9^/L and elevated liver enzymes with AST up to 248 U/L, ALT up to 263 U/L, Alkaline phosphatase (ALKP) up to 254 and Gamma-glutamyltransferase (GGT) up to 168 IU/ml. A skin biopsy performed that was consistent with DRESS (Supplement Fig.2 E, F). Laboratory investigations were negative for viral serology: EBV, CMV, HIV, HAV, HBV, HCV, Human T-cell lymphotropic virus type 1 (HTLV1), Human T-cell lymphotropic virus type 2 (HTLV2), negative polymerase chain reaction for HHV6, mycoplasma and SARS-CoV2, and negative immunological serology for anti-nuclear antibodies (ANA), anti-mitochondrial antibodies (AMA) and anti-parietal antibodies. Protein electrophoresis was negative for paraprotein, and immunoglobulin levels were within normal limits. In an attempt to minimize the use of systemic steroids in this patient with pathological fracture and active infection, following informed consent and committee approval of off-label drug administration, the patient received 30 mg SC injection of benralizumab. During following days, the patient developed anemia that was initially presumed to be autoimmune and she was started i.v. hydrocortisone. Gastroscopy performed, revealed a large gastric ulcer, and therefore, in addition to initiation of PPI, hydrocortisone was converted to prednisone 40 mg. During the acute phase, serum soluble IL-2 receptor was profoundly elevated (48,000 U/mL) and HLH secondary to DRESS was suggested. CT revealed hepatosplenomegaly with no evidence of lymphadenopathy or neoplasm. Further hematological workup was negative for BCR-ABL and JAK-2 mutation as well as T and B cell receptor rearrangement. The patient clinical symptoms and laboratory anomalies improved drastically following treatment (Supplement Fig. 2C, D). Follow-up serum soluble IL-2 receptor levels decreased to 6000 U/mL and hence bone marrow biopsy was not performed. One week following benralizumab treatment, the patient was discharged home with 30 mg prednisone with recommendation to rapid taper off therapy, 5 mg decline every 5 days. Following discharge, gastroscopy results were positive for *Helicobacter pylori*, and the patient received antibiotic treatment accordingly. Out clinic follow up four months following benralizumab therapy was unremarkable, with no recurrence of symptoms and complete resolution of laboratory deviations.
