# Supplementary material for: Novel targeted inhibition of the IL-5 axis for drug reaction with eosinophilia and systemic symptoms syndrome
Source: Front Immunol. 2023 Apr 28;14:1134178. doi: 10.3389/fimmu.2023.1134178 (PMC10175640; doi:10.3389/fimmu.2023.1134178)
Supplement: Supplementary file 2 [file Table_1.docx]

| **Items*** | No | Yes | Unknown |
| --- | --- | --- | --- |
| **Fever** ≥ 38.5ºC | -1 | 0 | 1 |
| **Enlarged lymph nodes** (≥2 sites, >1 cm) | 0 | 1 | 0 |
| **Atypical lymphocytes** | 0 | 1 | 0 |
| **Eosinophilia** –  700-1,499 (cells per mL) or 10%-19.9%  ≥1,500 or ≥ 20% | 0 |  | 0 |
|  |  | 1 |  |
|  |  | 2 |  |
| **Skin rash -**  Extent > 50% body surface area  At least 2 of: facial edema, infiltration, purpura, scaling, psoriasiform desquamation  Skin biopsy suggestive of DRESS | 0 |  | 0 |
|  |  | 1 |  |
|  | -1 | 1 | 0 |
|  | -1 | 0 | 0 |
| **Internal organ involvement**  1  ≥2 | 0 |  | 0 |
|  |  | 1 |  |
|  |  | 2 |  |
| **Rash resolution in ≥ 15 days** | -1 | 0 | 1 |
| **≥ 3 negative biological investigation to exclude other diagnosis*** | 0 | 1 | 0 |

**Supplementary table 1: RegiSCAR group study criteria**

.* Adopted from the Prospective RegiSCAR study , Kadaun et al. (4). Definite, probable, possible, and excluded DRESS diagnoses are defined by RegiSCAR scores of ≥6 , 4 - 5, 2-3 and <2 , respectively (4).

ANA: anti-nuclear antibody, HAV: hepatitis A virus, HBV: hepatitis B virus, HCV: hepatitis C virus, Mycoplasma, Chlamydia, blood cultures.
